# Finding the Missing Patients With Tuberculosis: Lessons Learned From Patient-Pathway Analyses in 5 Countries

**DOI:** 10.1093/infdis/jix388

**Published:** 2017-11-06

**Authors:** Christy Hanson, Mike Osberg, Jessie Brown, George Durham, Daniel P Chin

**Affiliations:** 1 Macalester College, St. Paul, Minnesota; 2 Bill and Melinda Gates Foundation; 3 Linksbridge, Seattle, Washington

**Keywords:** Tuberculosis, patient-pathway analysis, care seeking, private sector

## Abstract

**Background:**

Despite significant progress in diagnosis and treatment of tuberculosis over the past 2 decades, millions of patients with tuberculosis go unreported every year. The patient-pathway analysis (PPA) is designed to assess the alignment between tuberculosis care-seeking patterns and the availability of tuberculosis services. The PPA can help programs understand where they might find the missing patients with tuberculosis.

**Methods:**

This analysis aggregates and compares the PPAs from case studies in Kenya, Ethiopia, Indonesia, the Philippines, and Pakistan.

**Results:**

Across the 5 countries, 24% of patients with tuberculosis initiated care seeking in a facility with tuberculosis diagnostic capacity. Forty-two percent of patients sought care at level 0 facilities, where there was generally no tuberculosis diagnostic capacity; another 42% of patients sought care at level 1 facilities, of which 39% had diagnostic capacity. Sixty-six percent of patients initially sought care in private facilities, which had considerably less tuberculosis diagnostic capacity than public facilities; only 7% of notified cases were from the private sector. The GeneXpert system was available in 14%–41% of level 2 facilities in the 3 countries for which there were data. Tuberculosis treatment capacity tracked closely with the availability of diagnostic capacity. There were substantial subnational differences in care-seeking patterns and service availability.

**Discussion:**

The PPA can be a valuable planning and programming tool to ensure that diagnostic and treatment services are available to patients where they seek care. Patient-centered care will require closing the diagnostic gap and engaging the private sector. Extensive subnational differences in patient pathways to care call for differentiated approaches to patient-centered care.

Adopted by the member states of the United Nations in 2000, the Millennium Development Goals called for 50% reductions in tuberculosis prevalence and mortality by 2015 (Goal 6, Target 8) [1]. The World Health Organization (WHO) reported that, between 1990 and 2015, tuberculosis mortality declined by 47% globally and that prevalence declined by 42% [2]. Countries, supported by the WHO, the donor community, and technical partners, intensified efforts to effectively diagnose and treat tuberculosis, helping to save an estimated 49 million lives [3]. Building on this progress, the Sustainable Development Goals, adopted in 2015 as the successor to the Millennium Development Goals, seeks to “ensure healthy lives and promote well-being for all at all ages” (Goal 3). Goal 3 of the Sustainable Development Goals specifically establishes the goal of ending the tuberculosis epidemic by 2030 [4].

Despite the progress in recent decades, tuberculosis remained one of the 10 leading causes of death in 2015 [5]. Tuberculosis is generally a curable disease; to reduce the burden of disease, it is imperative that all patients with tuberculosis have access to diagnosis and treatment. Of the 10.4 million incident cases of tuberculosis in 2015, approximately 6 million were notified to national tuberculosis programs across the world [2]. This implies that there is a significant population of patients with tuberculosis who go unidentified (hereafter referred to as “missing”). It is essential to find these 4 million missing patients and to ensure that they receive quality care. The End TB Strategy of the World Health Organization emphasizes patient-centered care; patients should be able to access appropriate and affordable health services where they live [6].

Recognizing the challenge of locating the missing patients with tuberculosis, we performed an assessment of barriers to the reduction of the tuberculosis incidence in countries with a high tuberculosis burden, using the patient-pathway analysis (PPA). The PPA aims to identify common systemic barriers impeding patients’ ability to access diagnostic and treatment services, based on patient care-seeking patterns. This analysis summarizes and compares 5 country case studies that are presented elsewhere in this supplement.

## METHODS

We review, compare, and discuss results from 5 country case studies that used the PPA methods to assess the alignment of tuberculosis service delivery with patient care-seeking patterns [7]. The country case studies are from Ethiopia, Indonesia, Kenya, Pakistan, and the Philippines.

The PPA methods use available tuberculosis-specific, general care-seeking, and health systems data to understand the alignment of service delivery to patient behavior. In general, the sources of data include (1) population-based surveys, including census, Demographic and Health Surveys, living standards measurement surveys, health expenditure and utilization surveys; (2) health service availability assessments, including human immunodeficiency virus (HIV)/AIDS service provision assessments, service availability and readiness assessments, and health facility inventories; (3) government disease surveillance; (4) published literature; (5) government plans, reports, and proposals to the Global Fund; and (6) partner-initiated reports, such as technical support missions and program reviews.

In the country case studies, we used the most recently available data and studies and only included references published or data collected after 2005. In all cases, we preferred population-based, tuberculosis-specific data to general care-seeking data. However, general care-seeking data were used as a proxy for tuberculosis-specific care-seeking data when tuberculosis-specific information was not available or sufficiently detailed. The relevance of general care seeking as a proxy for tuberculosis-specific care seeking was confirmed in several of the country studies when multiple sources of data were available. Raw data were pulled and analyzed from the Demographic and Health Surveys and other available population-based surveys.

The constitution of the health system is different in each country. The roles of private and nongovernmental organization sectors vary, as do the structure of health system levels. To facilitate comparison across countries, we classified health facilities as either public or private (including nongovernmental organizations) and categorized them into the following service delivery levels: level 0 (L0), for community-based care; level 1 (L1), for primary health centers and clinics; and levels 2 and 3 (L2/L3), for secondary and tertiary care hospitals. In the accompanying Kenya case study, the health system levels are different than the standard categories because it uses the country’s own categorization system. For the purpose of these results, the health levels used in Kenya were translated to the common levels used throughout the rest of the case studies. This mapping is shown in Table 1.

**Table 1.** Mapping of Kenya Health Facility Levels to Standard Levels

Kenya Level	Kenya Facility Type	Standard Level
5	Teaching and referral hospitals	3
4	General hospitals	2
3	Primary care clinics	1
2	Dispensaries/pharmacies/shops	0
1	Community health worker/traditional healer facilities	0

This analysis compared the findings from individual countries and developed conclusions based on an aggregation of the pathway data across the 5 countries for which patient-pathways were completed. To aggregate data from the 5 country pathways, the individual country care-seeking patterns were multiplied by the estimated tuberculosis burden of the country to give more-populous countries greater weight in the overall care-seeking percentages. Therefore, the overall care-seeking patterns were weighted more heavily toward Indonesia and Pakistan because these countries were estimated by the WHO to have more patients with tuberculosis than the other countries [2].

We lacked data for the availability and placement of radiography, the GeneXpert system, and culture in several countries. However, we had data on the availability of microscopy services for all countries. As such, we use the availability of microscopy as a proxy indicator for understanding the alignment between patient care seeking and the availability and distribution of tuberculosis diagnostic technologies. While the future of tuberculosis elimination will rely on more-advanced diagnostic technologies than simple microscopy, we can learn from the intensive scale-up of laboratory capacity for microscopy that has occurred in most countries with a high tuberculosis burden over the past 20 years. Many countries that have expanded diagnostic capacity through microscopy continue to have persistent gaps in patient access. We can study the introduction and expansion of microscopy in these countries to understand how best to introduce and distribute emerging technologies, as well as to identify necessary supporting systems, such as specimen transport, that are needed to optimize the use of these technologies.

## RESULTS

In 2015, the 5 countries had 25% of the estimated global tuberculosis incidence, 34% of the estimated tuberculosis incidence among HIV-positive people, 18% of the incidence of multidrug-resistant (MDR)/rifampicin-resistant tuberculosis, and 28% of the missing tuberculosis cases worldwide [2]. In each country study, the patient-pathways analysis sheds light on misalignment between the location of patient care initiation and the location of available diagnostic and treatment services.

### 24% of Tuberculosis-Specific Care Seekers Encountered a Health Facility With Diagnostic Capacity at Their First Visit

**Figure 1.**
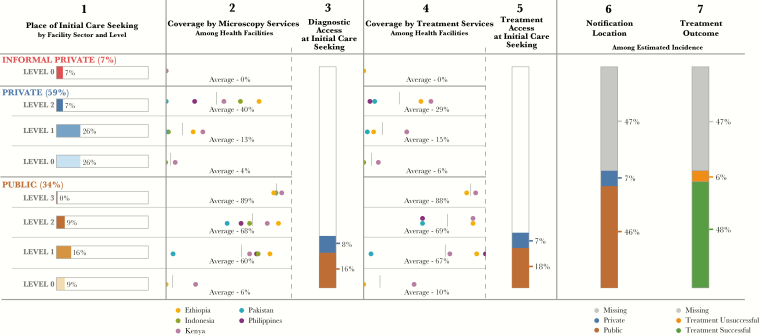
Patient-pathway visual—5-country summary. The 5-country patient-pathway visual combines data from 5 of the country profiles included in the accompanying supplement (Ethiopia, Indonesia, Kenya, Pakistan, and the Philippines). Column 1 of the pathway shows cumulatively the place of initial care seeking across all 5 countries. This was calculated by multiplying data on initial care seeking in each country at the respective sectors and levels by the estimated incidence of tuberculosis in that country. The resulting care-seeking values were then summed across country, sector, and level to estimate approximate care-seeking patterns for all 5 countries. Columns 2 and 4—diagnostic and treatment coverage—were calculated using the average coverage among countries with available data. Only data on microscopy and treatment provision were used for this summary pathway, because these were the data points most widely available across all 5 countries. In cases where data was missing for any country, coverage was averaged across the remaining country data points, with the exception of L0 facilities (all sectors), for which microscopy and treatment coverage was assumed to be 0% in countries without data. Columns 3 and 5 were calculated by multiplying the share of care seeking at each sector and level by the average coverage of diagnosis and treatment services, respectively, at each sector and level. The results were then aggregated across public and private sectors to provide estimates of the percentage of patients likely to access diagnosis and treatment on their initial visit to a healthcare facility. Column 6 shows the location of case notification for cases notified to the World Health Organization (WHO). Notification location is calculated among the total estimated burden of the 5 countries profiled, and nonnotified cases are labeled as “missing.” Column 7 shows treatment outcomes among patients notified to the WHO. Treatment success rates from the 2016 WHO report for each country were evaluated with respect to their notified cases to calculate the number of patients who did and those who did not successfully complete treatment. These numbers were then calculated among the total estimated burden of the 5 countries profiled, with nonnotified cases labeled as “missing.”

Figure 1 presents the aggregate 5-country patient pathway, showing the alignment between patient care-seeking patterns and tuberculosis services. The first 2 columns provide the patterns of care initiation (column 1) and microscopy coverage (column 2), by health facility sector and level. Multiplication of data in these 2 columns, aggregated across all health facility types, provides the result for the third column, which shows access to diagnosis at the point of initial care. This result shows the percentage of patients who initiate care in a facility that has tuberculosis diagnostic capacity.

Across these 5 countries, 24% of patients (range, 5%–45%) initiated care in health facilities that had tuberculosis microscopy services available (laboratory records and Xpert test records, National Tuberculosis, Leprosy, and Lung Disease Unit [NTLD], Kenya Ministry of Health [MOH], personal communication, 2016) [8–14]. Thus, the remaining 76% of these patients encountered a health facility that did not have diagnostic capacity. However, some of these facilities could have a system to refer patients or their sputum specimens to another facility for diagnosis. Although there was evidence in several countries that referral systems were in place for both patients and samples, there were insufficient data to determine the proportion of facilities that had the capacity for such referrals.

In all countries except Pakistan, >50% of the public health centers (L1) and public hospitals (L2) had smear microscopy [9–13]. However, for all countries, smear microscopy was available in <50% of facilities where patients initiated care. This was due to the popularity of lower-level providers and private-sector facilities for care initiation [14–20].

### COMMON FINDINGS AMONG CASE STUDIES

The case studies focused on each level of the health system, by sector, to highlight misalignments in patient care initiation and tuberculosis care provision and to underscore the importance of patient-centered planning. The analyses yielded several common findings across countries.

#### L0 Facilities Tended to Have No Diagnostic Capacity and Insufficient Referral Programs

L0 health facilities were important entry points for patients in all 5 countries. In Ethiopia and the Philippines, public-sector community health workers represented the locus of care initiation for nearly a third of patients [17, 20]. However, community health workers were prepared not to diagnose tuberculosis, but rather to screen, collect sputum specimens, or refer patients presumed to have tuberculosis. In Kenya, L0 public dispensaries were the initial point of care for 27% of patients, and 20% could conduct microscopy (laboratory records, NTLD, Kenya MOH, personal communication, 2016) [15]. However, the majority needed to refer patients for diagnosis. While most countries have policies that require community health workers and dispensaries to refer patients from public-sector L0 facilities to L1/L2 facilities, there are insufficient incentives, enablers, and systems in place to enable consistent patient referral [2]. For example, none of the countries had nationwide sputum specimen collection at L0 facilities, but most countries had pilot projects for sputum specimen collection, staining, or transport at L0 facilities [2]. Similarly, there were nascent efforts to provide funding for patient transport, as well as for information systems that enable tracking of patients presumed to have tuberculosis [2].

In Indonesia and Pakistan, 52% and 24% of patients, respectively, initiated care in private (formal or informal) community-level health facilities (L0) [16, 18]. As is the case for public L0 facilities, private L0 pharmacies and drug shops do not have the capacity for quality tuberculosis diagnosis (laboratory records and Xpert test records, NTLD, Kenya MOH, personal communication, 2016) [8–13]. However, private L0 facilities were not subject to the same policies requiring screening, referral, or sample collection [21–25]. Consequently, a large proportion of the patients who initiated care at private L0 facilities were responsible for finding their own way to facilities with adequate tuberculosis diagnostic and treatment capacity, potentially delaying or permanently stalling the provision of appropriate care.

#### Many L1 Facilities Had No Diagnostic Capabilities

**Figure 2.**
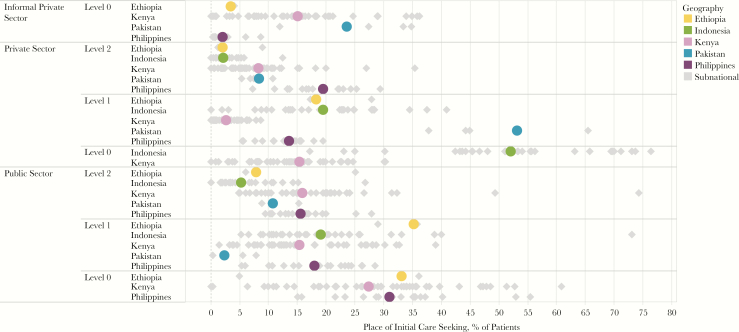
Care-seeking patterns across countries. Care-seeking patterns are diverse across countries, requiring programs tailored to the sectors and levels accessed by patients. Subnational care-seeking patterns (gray diamonds) show wide disparities within countries.

More than 40% of patients (range, 18%–55%) across all countries initiated care in L1 facilities (Figure 2; laboratory records and Xpert test records, NTLD, Kenya MOH, personal communication, 2016) [8, 9, 11, 12, 14, 19, 20]. Yet in aggregate, an average of 39% of L1 facilities (range, <1%–82%) had diagnostic capabilities (Figure 1) [9–13]. Patients visited private L1 facilities during 3%–53% of initial care visits, although only an average of 13% of these facilities (range, <1%–29%) had diagnostic capacity (data are for 4 countries; the Philippines did not have sufficient data to calculate coverage for L1 private-sector facilities) [9–13]. In Pakistan, 53% of patients initiated care in private L1 facilities, but only 1% of those facilities could conduct smear microscopy [19].

Use of public L1 facilities also varied widely, from 2% of patients in Pakistan to 35% of patients in Ethiopia [19, 20]. The inconsistency in diagnostic capacity across L1 facilities potentially represents disparities in access, as well as an unpredictable quality of care for patients.

#### Diagnostic Capacity Was Strongest but Care Initiation Limited at L2/L3 Facilities

Patients were less likely to initiate care at the hospital level (L2/L3) than at more-decentralized facilities (L0 and L1). Between 2% and 20% of patients initiated care in private hospitals (Figure 2; laboratory records and Xpert test records, NTLD, Kenya MOH, personal communication, 2016) [8, 9, 11, 12, 14, 19, 20]. Despite the limited use of private hospitals by patients with tuberculosis in most countries, the majority of hospitals had diagnostic capacity [9–13]. In Indonesia, for example, 73% of public hospitals had microscopy services, while only 5% of patients initiated care there [12]. Similarly, in Ethiopia, 89% of public hospitals had microscopy services, and around 80% had radiography services [13], but only 8% of patients initiated care in those facilities [20]. It becomes obvious from the PPA that the real usefulness of diagnostic capacity at L2/L3 facilities is in the care of patients referred from lower levels of the system. If there are effective systems in place to ensure the referral of patients and samples, then it will be easier to capitalize on the diagnostic capacity of L2/L3 facilities.

#### Private Sector Engagement Remains an Important Challenge

**Figure 3.**
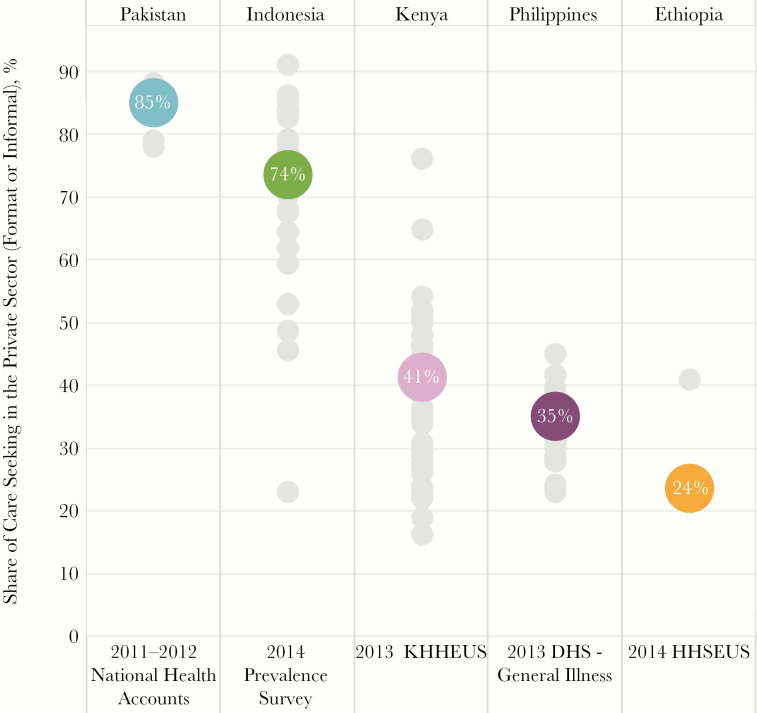
Private-sector care-seeking patterns. Private-sector healthcare facilities play an important role across all countries. This visual shows the share of patients seeking care in either formal or informal private-sector care facilities. At the national level (colored circles), 74% of patients in Indonesia and 85% of patients in Pakistan initiate care seeking in the private sector. However, private sector care-seeking patterns at sub-national levels (gray circles) in countries such as Indonesia (range, 23%–91%) and Kenya (range, 16%–76%) vary widely. Abbreviations: HHSEUS, Household Health Services Utilization Expenditure Survey; KHHEUS, Kenya Household Health Expenditure and Utilisation Survey.

An estimated 66% of patients (range, 24%–85%) initiated care in the private sector (Figure 3; laboratory records and Xpert test records, NTLD, Kenya MOH, personal communication, 2016) [8, 9, 11, 12, 14, 19, 20]. In 2015, only 13% of cases (range, 6.4%–22%) notified to national tuberculosis programs came from private-sector providers [2, 3, 18, 24, 26]. Given that, many patients are likely receiving a tuberculosis diagnosis and treatment in the private sector and not notified. Alternatively, they may make multiple care visits and end up in the public sector to receive care for tuberculosis.

It is noteworthy that the private-sector care-seeking patterns were quite heterogeneous within countries, suggesting that public-private mix activities need to be differentiated to match these patterns (Figure 3). For example, in Indonesia, 74% of patients sought care in the private sector at the national level, but this ranged from 23% to 91% of patients across the provinces [16]. Nationally, in Kenya, 42% of patients initiated care in the private sector, but at the county level, this ranged from 16% to 75% of patients [14, 15].

Countries also need to consider the level at which patients engage with the private sector. The level of private care most heavily utilized and the diagnostic and treatment capabilities at these facilities varied widely between countries and subnationally. Only in the Philippines did >10% of care initiations occur in private hospitals. Most private-sector utilization was at L0 and L1 facilities, such as pharmacies (L0) and general practitioner facilities and clinics (L1) [17]. In Pakistan, for example, nearly 80% of patients initiated care with private providers in L0 and L1 facilities [19]. The government has responded by collaborating with >3500 private practitioners to ensure the delivery of quality tuberculosis care [10].

In some countries, the paucity of data on the private sector reflects the absence of regulation of this sector. Public health programs often have little visibility of the activities of private providers and thus cannot ensure quality care for patients with tuberculosis seeking care there. As the case studies suggest, mandatory notification is needed from private-sector providers, but this needs to be coupled with strong enforcement, along with incentives and enablers, to ensure compliance.

#### Treatment Availability Is Slightly Better Aligned with Patient Care-Seeking Preferences

In aggregate, 25% of patients initiated care in sites that had tuberculosis treatment services. This was only slightly better than initial access to a tuberculosis diagnostic technology. However, when we looked at the location of case notification and reporting of treatment outcome, we saw a different story. While 34% of patients initiated care in the public sector, 46% of all estimated cases of tuberculosis were notified by public-sector facilities. At the country level, it appeared that the proportion of patients receiving treatment at each level of the health sector mirrored the proportion who initiated care in the respective facility levels but with expanded use of the public sector for treatment as compared to initial care seeking. This suggests that patients had to move to the public sector for treatment. In many countries with decentralized treatment services, the results showed a higher proportion of patients being treated at lower levels—commonly commensurate with initial care seeking—than should have been the case, given the low reported availability of tuberculosis treatment at that level. This can be explained by the fact that in many countries, antituberculosis medicines are only distributed to lower-level facilities when a patient is notified (ie, no supplies are maintained at lower levels). The surveys that captured service availability data recorded tuberculosis treatment availability only if medicines were in stock in the facility on the day of the survey. In Ethiopia, for example, 76% of patients initiated care in the public sector, and only 40% accessed a facility with tuberculosis medicines available. However, an estimated 68% of incident cases received treatment in the public sector [13, 20]. While treatment success among notified cases was high, outcomes were only reported for 54% of patients. As such, only 48% of all estimated patients with tuberculosis were known to have been successfully treated.

#### Extensive Subnational Differences in the Patient Pathway Call for Differentiated Approaches

**Figure 4.**
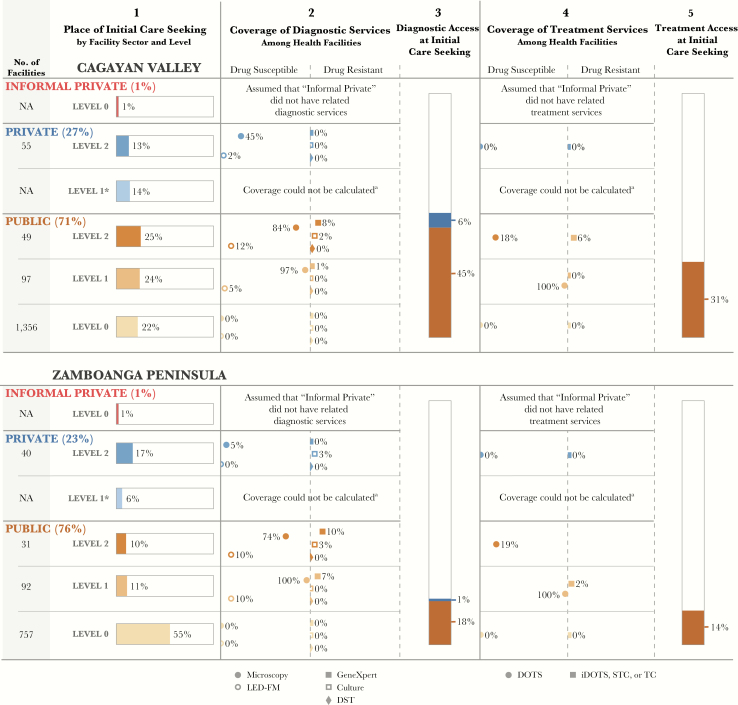
Comparison of subnational pathways in 2 provinces in the Philippines. When the patient pathway is completed at the subnational level, it can highlight important differences in care seeking and service alignment patterns of national tuberculosis programs. This visual highlights 2 regions in the Philippines with important differences in the alignment of care-seeking patterns and service coverage. The top visual shows Cagayan Valley (Region II), which has relatively uniform care seeking across the 3 levels of the public-sector healthcare system. Owing to the high coverage by diagnostic tools at levels 1 and 2, >50% of patients are likely to access diagnosis on their first visit to a health facility (column 3). The bottom visual shows the pathway for Zamboanga Peninsula (Region IX). In this region, more than half of patients initiate care seeking at level 0 public sector facilities, where coverage by tuberculosis diagnostic tools is not available. Thus, these patients are reliant on a referral system to receive a diagnosis for tuberculosis. Owing to the higher share of patients seeking care where diagnostic tools are not available, the access to diagnosis at initial care seeking metric (column 3) is much lower in Region IX. Abbreviations: DOTS, directly observed therapy, short course; DST, drug-susceptibility testing; iDOTS, integrated directly observed therapy, short course; LED-FM, light-emitting diode fluorescence microscopy; NA, not available; STC, satellite treatment center; TC, treatment center. ^a^The National Health Facility Registry does not provide reliable data on the number of health facilities in private-sector level 1 facilities.

In all countries, we noted intracountry differences in the patient-pathways (ie, the dynamic between patient care seeking and system capacity varied across countries). Figure 4 presents an example from the Philippines. In one region, Zamboanga Peninsula, more than half of patients initiated care with public providers at the community level (L0) [17]. These community health facilities provided no diagnostic services. The national policy calls for community health workers to identify and refer patients to L1 rural health units [23], where 71% of facilities have microscopy capability [8, 9]. This referral network, involving remote sputum specimen collection and staining, is important for patients in this region. In Cagayan Valley, care initiation was primarily distributed across levels of the public sector [23]. While private-sector hospitals (L2) in this region had more diagnostic capacity than those in Zamboanga Peninsula [7, 9], the difference in the availability of diagnostic capacity at the site of care initiation was largely driven by differences in care-seeking patterns in the public sector [23].

**Figure 5.**
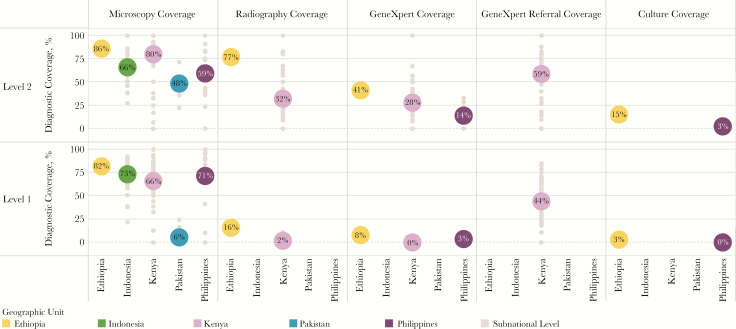
Diagnostic coverage among public sector primary care facilities and hospitals. This visual compares the coverage of diagnostic services among public sector level 1 (primary care) and level 2 (hospitals) health facilities. Colored circles indicate coverage of diagnostic tools at the national level, and gray circles indicate coverage in respective subnational levels (eg, regions in the Philippines, provinces in Indonesia and Pakistan, and counties in Kenya).

Figure 5 displays the wide variance of diagnostic availability within the public sector across countries. Consider the example of Kenya, which has one of the highest rates of access to diagnostic tools at a national level, as well as the widest range of diagnostic coverage across its counties (laboratory records and Xpert test records, NTLD, Kenya MOH, personal communication, 2016) [11]. This suggests relatively efficient allocation of technologies to facilities that have higher patient loads. However, it also signals intracountry disparities across the country that could help explain why patients with tuberculosis are missed. On a subnational level, analysis revealed that access to any diagnostic service ranged from a low of 17% of patients in one county in Kenya, to a high of 79% in another (laboratory records and Xpert test records, NTLD, Kenya MOH, personal communication, 2016) [11, 15].

In some countries, key differences emerged between urban and rural areas. In Kenya’s urban areas, for example, 36% of initial care episodes occurred in private facilities, while in rural areas, only 22% of patients sought care in the private sector [14, 15]. In urban areas, care initiation in the public sector was primarily in hospitals (33% of cases), while rural populations sought care in public primary care facilities (35% of cases) [14, 15].

### Utilization of the Xpert System and Chest Radiography

Most countries reported that they are revising or have already revised their diagnostic algorithms to include the use of Xpert as the initial diagnostic test for tuberculosis, as per the recently updated WHO guidelines for the programmatic management of tuberculosis [21–25]. In addition, all countries used Xpert to detect rifampicin resistance and to screen for MDR tuberculosis [2]. However, access to Xpert at the point of care initiation was extremely limited in the 3 countries for which data were available. In Ethiopia, Kenya and the Philippines, Xpert was available in 41% [13], 28% (Xpert test records, NTLD, Kenya MOH, personal communication, 2016), and 14% [17] of public hospitals, respectively. Xpert was available in only a limited number of L1 facilities (Xpert test records, NTLD, Kenya MOH, personal communication, 2016) [13, 17, 27]. Decentralization of Xpert availably to L1 facilities is not routinely recommended, given its operating requirements, expense, and potential for underutilization. Even with the Xpert system’s current positioning in centralized laboratories, a previously published review of the average number of Xpert cartridges used per module suggests a dramatic underutilization of the existing technology [28]. The misalignment with care seeking reinforces the key finding that specimen transport systems will be paramount to increase timely access to diagnosis overall and specifically for the utilization of Xpert for case identification.

Data on the availability of radiography were only available for Ethiopia and Kenya. In Ethiopia, approximately 16% of patients had access to radiography where they initiated care [11], while 23% of notified tuberculosis cases were clinically diagnosed [2]. In Kenya, radiography was available for approximately 10% of patients, and 25% of all notified cases were clinically diagnosed [11]. We did not have data on the likelihood of referral to radiography facilities in support of clinical diagnosis.

## DISCUSSION

### Focus on Patient-Centered Care

This review, along with the accompanying country case studies, points to some of the systemic barriers to finding the world’s 4 million missing patients with tuberculosis. By paying attention to the gaps in diagnosis and treatment, we can gain insights into the whereabouts of missing patients with tuberculosis, particularly those who actively but unsuccessfully seek care. The intent of the PPA is not to determine how patients should change their care-seeking behavior to access tuberculosis services. Rather, it is to determine how to deliver patient-centered care. Addressing the gaps in the patient pathway is the way to achieve patient-centered care; this provides solutions that will deliver necessary services to patients where they are. It enables access to quality services via patients’ preferred providers, at the level that is most accessible, affordable, and appropriate for them. Better understanding of the points of misalignment between current tuberculosis service availability and how patients seek care can guide programmatic priorities and interventions. As new technologies become available, the PPA can inform not only the most efficient placement of the technology, but also the systems needed to optimize their utilization.

Across the 5 countries, three quarters of initial care visits occurred in L0 and L1 facilities [14–20]. This points to the importance of closing the diagnostic gap in these decentralized facilities. There are several reasons why it is most important to establish diagnostic capabilities in L1 facilities. First, >40% of patients with tuberculosis initiate care at these facilities. Second, L1 facilities are the closest facilities for referral of patients or specimens from L0 facilities, where another one third of patients initiate care. Third, if patients can get a tuberculosis diagnosis in L1 facilities, this obviates the need to go to a L2 or L3 facility for diagnosis. However, only 60% of public and 13% of private L1 facilities have the capacity for microscopy (laboratory records, NTLD, Kenya MOH, personal communication, 2016) [8, 11–13, 29]; there is a huge gap in diagnostic services that needs to be closed. With almost no diagnostic capacity at L0 (laboratory records and Xpert test records, NTLD, Kenya MOH, personal communication, 2016) [8, 11–13, 29], there is a need to establish systems to refer patients or sputum specimens to L1 or L2 facilities and to improve information flow back to L0 facilities so that patients and services are better connected.

To improve the diagnostic capacity at L1 facilities, one can establish a specimen referral and transportation network from these facilities to a central laboratory at a L2 facility. Alternatively, one can place additional diagnostic tools into L1 facilities. The Xpert Omni, when it becomes available, is a molecular diagnostic system with the potential for use in L1 facilities. However, the feasibility of using Omni or any other molecular test at this level has yet to be demonstrated. Radiography can also be introduced at this level as a triage test to identify those who need referral or testing.

We were surprised to see that diagnostic capacity was incomplete among hospitals (L2), as <70% of public hospitals and only around 40% of private hospitals had equipment to diagnose tuberculosis (laboratory records and Xpert test records, NTLD, Kenya MOH, personal communication, 2016) [8, 11–13, 29]. This gap should be urgently closed. Until it is shown that a point-of-care molecular test for tuberculosis can be used in L1 facilities, molecular testing is only feasible at L2 or L3 facilities. L2 facilities are the ideal hub for the hub-and-spoke laboratory model, accepting specimen referrals from L1 facilities. But this will require a system to rapidly transport specimens and provide laboratory results back to the originating facilities.

Finally, the PPA affirms the importance of decentralized planning. The PPA has shown that patients’ care-seeking behavior and the availability of health facilities are heterogeneous. Therefore, in different countries and different parts within a country, there are likely different ways to build a laboratory network that facilitates patient-centered care. Most countries operate in a budget-constrained environment. Countries should use data such as those from the PPA to determine exactly where it makes the most sense to roll out and place new diagnostic technologies.

### Differentiated Planning for the Engagement of the Private Sector Is Essential

This analysis confirmed that in many countries, the private sector is the dominant source of initial care. On average, 66% of patients initiated care in the private sector [14–20]. However, only a small percentage of patients who were notified to national tuberculosis programs (NTPs) were being treated in the private sector. This may mean that many of those initiating care in the private sector had to be seen by several providers before receiving diagnosis and treatment in the public sector by programs linked to the NTPs. Alternatively, many are treated in the private sector—frequently poorly—and are never reported to NTPs. In countries where the majority of patients with tuberculosis initially seek care in the private sector, activities that engage the private sector are particularly central in the quest to find missing tuberculosis cases.

However, it is not clear to what extent many of the current public-private mix tuberculosis strategies directly respond to the context-specific patient care-seeking preferences. In Indonesia, over half of all care seeking is initiated in L0 private pharmacies and drug shops [16]. Meanwhile, in the Philippines, 20% of care seeking is initiated in private hospitals [17, 18]. The priorities and model(s) to appropriately engage health providers in these 2 countries should be vastly different. In addition, subnational heterogeneity of private-sector capacities and utilization further warrant highly differentiated approaches to optimize the engagement of private providers, particularly through the focusing of resources where they will reach the most patients early in the care-seeking continuum.

### Quality Treatment Services Are Limited to the Public Sector

Treatment services were better aligned with patient care-seeking patterns than with diagnostic services among the 5 countries analyzed, but treatment was still limited in important locations where patient seek care. Across all levels in the formal private sector, for example, <30% of facilities had treatment services available [8, 11, 13, 16, 29]. Several studies suggest that tuberculosis drugs are used widely in the private sector, but the quality of these drugs, as well as monitoring and support services, are mostly unknown [30].

Treatment success among notified cases remains high and notifications are mostly provided by public sector facilities [4, 31–33]. As countries continue to find more of the missing cases, maintaining the same level of treatment success with limited budgets may require innovative ways of supporting the quality of care for patients across diverse settings, including in the private sector. Updated WHO treatment guidelines were released in 2017, recommending that community-based or home-based directly observed therapy be pursued over facility-based directly observed therapy and that lay and community health workers be further engaged with treatment administration and support. Additionally, recommendations were included for using new monitoring technologies, such as medication monitor or video directly observed treatment to support patients receiving treatment [34]. These recommendations align well with the findings of the PPA, as the patients who initiate care at L0 facilities likely prefer to receive care at that level (ie, close to home).

### MDR Tuberculosis Services Need Continued Scale-up

In 2015, only 24% of bacteriologically confirmed new cases and 53% of retreatment cases globally underwent drug-susceptibility testing for rifampicin. There is a great need to scale up patient-centered testing for MDR tuberculosis [2]. Unfortunately, the availability of services to diagnose MDR tuberculosis remains low. Data on Xpert coverage from Ethiopia, Kenya, and the Philippines showed that Xpert was available in <10% of public L1 facilities and in 14%–41% of public L2 facilities (Xpert test records, NTLD, Kenya MOH, personal communication, 2016) [13, 17, 27]. There was little evidence that private-sector facilities had coverage of Xpert.

The PPA showed that there is a significant gap between where patients initially seek care and facilities that can provide the drug-resistance testing. Thus, patients have to move between many facilities to get tested. However, in Kenya, a system linking facilities to centralized Xpert testing facilities by use of a referral network actually boosted the proportion of patients testing for drug-resistant tuberculosis (Xpert test records, NTLD, Kenya MOH, personal communication, 2016). Given the high proportion of patients initially seeking care in the private sector, there is a need for models to engage the private sector with referral options for Xpert testing [14–20].

It is encouraging that first-line drugs and treatment support were widely available through the public sector [8, 11, 13, 16, 29], and availability was relatively consistent with patient care-seeking preferences [14–20]. Decentralization of treatment capacity for MDR tuberculosis should be similarly pursued to enable patient-centered care. Kenya provides a good example of this. As patients from the catchment area of a particular L1 facility receive a diagnosis of MDR tuberculosis, the program builds the capacity of the L1 facility to provide MDR tuberculosis care. Thus, MDR tuberculosis treatment is scaled up in lock step with expanded diagnosis [2].

### Using the PPA for Planning and Programming

Obviously, PPA is not sufficient, in itself, to guide programmatic priorities. The PPA should complement other tools, including other quantitative and qualitative inputs, to build a suite of evidence. The WHO recommends that countries administer periodic tuberculosis prevalence surveys until routine tuberculosis surveillance is sufficiently robust that population-based surveys are no longer needed as a means of assessing the tuberculosis burden. The WHO also recommends periodic epidemiological reviews and external monitoring missions to facilitate objective reflections on programmatic strengths and challenges, as well as to promote the effective distribution of review findings [2]. Analysis and interpretation of programmatic data are also essential for decision-making. Increasingly, modeling of cost-effectiveness and the impact of possible interventions is available at a national level. All of these inputs together comprise a robust evidence base for programmatic priority setting and planning.

Each PPA, whether national or subnational, will require local interpretation. Interpretation should incorporate the inputs of other evidence, as noted above, as well as local knowledge. When a PPA reveals an apparent misalignment between where patients seek care and where services are available, countries may already have solutions in place that bridge the identified gap. In rural Ethiopia, for example, the PPAs showed high levels of care seeking at L0 health facilities [20]. While this might seem problematic, based on data showing low diagnostic capacity in L0 facilities [13], this finding itself is not necessarily bad. In Ethiopia, the design of the health system has made community-based health extension workers available to the entire rural population. The health workers operate at the community level and are systematically networked to health facilities that, to a large extent, have tuberculosis diagnostic capacity, thus mitigating concerns about low access to diagnosis at L0 facilities [35].

### Expanded Application of PPAs Will Require Standardized Data

The availability of population-based survey and programmatic data was essential to developing the PPAs described in this supplement. While most countries have some assortment of these data sets, indicators varied widely, depending upon the survey or data collection platform. It is thus important to note that all cross-country conclusions are made using variable data and indicators. As the global tuberculosis community advances toward more-detailed, evidence-based programming, PPA findings will be an essential input. To do this most effectively and accurately, we must establish a set of standardized indicators that can be collected across survey platforms.

Addressing the health system barriers to enable timely access to appropriate diagnosis and treatment can be considered low-hanging fruit. In different ways, each country experience demonstrates the importance of considering the care-seeking preferences of patients as tuberculosis programs plan and implement interventions. The subnational patient pathways point to islands of excellence, where misalignment gaps have been addressed in support of patient-centered care, offering lessons from within countries themselves. The review yields a revised definition of diagnostic and treatment coverage that takes into account patient preference and proposes how to expand services in a manner that achieves patient-centered access.

## Notes

***Supplement sponsorship***. This work is part of a supplement sponsored by the Bill & Melinda Gates Foundation.

***Potential conflicts of interest.*** All authors: No reported conflicts of interest. All authors have submitted the ICMJE Form for Disclosure of Potential Conflicts of Interest. Conflicts that the editors consider relevant to the content of the manuscript have been disclosed.
